# Mineral Micronutrients in Asthma

**DOI:** 10.3390/nu13114001

**Published:** 2021-11-10

**Authors:** Dominika Zajac

**Affiliations:** Department of Respiration Physiology, Mossakowski Medical Research Institute, Polish Academy of Sciences, ul. Pawinskiego 5, 02-106 Warsaw, Poland; dzajac@imdik.pan.pl

**Keywords:** asthma, micronutrients, immunity, oxidative stress, inflammation, supplementation

## Abstract

Asthma represents one of the most common medical issues in the modern world. It is a chronic inflammatory disease characterized by persistent inflammation of the airways and disturbances in redox status, leading to hyperresponsiveness of bronchi and airway obstruction. Apart from classical risk factors such as air pollution, family history, allergies, or obesity, disturbances of the levels of micronutrients lead to impairments in the defense mechanisms of the affected organism against oxidative stress and proinflammatory stimuli. In the present review, the impact of micronutrients on the prevalence, severity, and possible risk factors of asthma is discussed. Although the influence of classical micronutrients such as selenium, copper, or zinc are well known, the effects of those such as iodine or manganese are only rarely mentioned. As a consequence, the aim of this paper is to demonstrate how disturbances in the levels of micronutrients and their supplementation might affect the course of asthma.

## 1. Introduction

Micronutrients are often referred to “vitamins and minerals, vital to healthy development, disease prevention and well-being” [1]. The six most important micronutrients are iron, vitamins A and D, iodine, folate, and zinc. Apart from vitamin D, these are not produced by the human organism and have to be delivered with food. The WHO list of essential medicines includes iodine, iron, zinc, and fluoride [2]. Other classifications of micronutrients include other vitamins and minerals such as copper, manganese, boron, selenium, molybdenum, cobalt, and chromium [3]. Prolonged deficiencies of these compounds lead to disturbances of various functions of the body such as anemia (in case of iron), dental malformations (in case of fluorine), or impaired immunity (zinc). One must always have in mind that some of these substances also have negative effects on the body, depending on their concentration, form (ionized or not), or even oxidation state (only the Cr (3+) ion has a positive effect). At the same time, due to environmental pollution, bad quality of food, and inadequate nutrition-related habits, more and more people in Western countries shows symptoms of microelement deficiency and tend to supplement them mostly in the form of pills sold under various brand names. Currently, additional intake of vitamin D, zinc, or selenium from nonfood sources is recommended [4]. Another big problem is malnutrition in middle-and low-income countries. According to the WHO, more than 200 million children under the age of five years suffer from malnutrition and its consequences, and the shortage of micronutrients such iron or iodine leads to disorders such as anemia, brain damage, mental impairment, blindness, or higher susceptibility to infections [5].

One of the biggest health problems of the modern world is an increased occurrence of disorders and diseases of the respiratory system. One of these disorders is asthma. It is a chronic inflammatory disease characterized by persistent inflammation of the airways, increased influx of inflammatory cells to the airways, and disturbances in redox status, all of which lead to hyperresponsiveness of the bronchi and airway obstruction. The exact case of asthma is not known. However, several risk factors such as family history, air pollution, and obesity have been described. Several phenotypes of asthma can be distinguished, depending on the clinical outcome (eosinophilia-related allergic asthma, neutrophilia-related non-allergic asthma, mixed form) or on the stimulus inducing an exacerbation (such as, among others, exercise-, aspirin-, and allergen-induced asthma) or the chronic state of the patients such as obesity or occupational factors including fumes, work with animals, latex, dust and many others, often overlapping with themselves [6].

In the last few years, various papers have been published indicating that in the course of asthma, decreased levels of micronutrients are observed [7,8,9,10]. The aim of the present paper is as a consequence to present the role of micronutrients in asthma.

Micronutrients themselves can be divided into three groups according to their impact on asthma. The first group includes micronutrients directly involved into immunity and as a consequence in the pathophysiology of asthma, namely copper, selenium, and zinc. The second cover minerals related to risk factors of asthma, such as chromium (obesity), iodine (disturbances of the thyroid gland), iron (anemia), and manganese (oxidative stress). The third group encompasses minerals of minor or unknown importance for asthma or its risk factors where the influence of their deficiency or overload remains only supposable.

## 2. Micronutrients of High Impact on Immunity and Major Features of Asthma

### 2.1. Copper

Copper (Cu) is, in humans, the third most abundant trace element. It is a component or cofactor of enzymes involved in energy metabolism (including cytochrome c oxidase), oxidant-antioxidant balance (including Cu-Zn-superoxide dismutase) or iron metabolism (including ceruloplasmin). Additionally, it takes part in the regulation of angiogenesis, response to hypoxia, and neuromodulation.

According to most researchers, copper levels are elevated in asthma [11,12,13], especially in women [14], even if no direct association between copper status and lung function can be found [15,16]. In general, both copper deficiency and excess can lead to chronic inflammation [15], and elevated Cu in serum, or parallel to decreased zinc and selenium levels or as increased copper-to-zinc and copper-to-selenium rations can be a marker of inflammation and of immune status. It is worth noting that low copper levels occur together with high zinc concentrations, and zinc deficiencies are observed parallel to copper excess [14]. This can result from the fact that increased copper levels inhibit Zn influx across intestinal membranes [17]. Increased copper levels may be due to copper release from tissue damage by inflammation [17]. Moreover, copper not only activates phosphatidyl-inositol-3-kinase (PI3K), an enzyme activating itself inflammatory mediators, inflammatory cell recruitment, and airway remodeling [18], but also stimulates IL-6 production and release [19,20]. As a consequence, copper ions might be some kind of second messenger in propagation of inflammation and response to inflammatory burden [21].

Ceruloplasmin (CP) is the main carrier protein for copper. It not only plays a role in iron metabolism but is also one of the components of the antioxidant defense, acting as a free radical scavenger. CP levels are elevated during oxidative stress [22] including the course of asthma [13]. Winter et al. [21] described that the increased levels of CP are rather a response to inflammatory burden as the highest levels of the protein were observed in asthma patients with systemic inflammation, severe or neutrophilic asthma.

Copper ions are necessary in the proper activity of the lysyl oxidase (LOX) responsible for the cross-linking of collagen and elastin. There is little known about LOX in asthma, where airway remodeling is one of the key features of the severe form of the disease. However, LOX activity is increased in fibrotic lung diseases such as idiopathic pulmonary fibrosis [22] but decreased in COPD, another inflammatory obstructive disorder of the airways [23], and LOX-deficient mice develop abnormal formation of bronchi with thick airway walls [24]. As LOX activity correlates with copper concentration, proper copper levels are supposed to be crucial for the correct function of the respiratory system [25].

Another important copper-containing enzyme is the Cu-Zn-superoxide dismutase (CuZnSOD), whose levels are decreased in asthma [26,27], likely due to the decreased levels of zinc [14] or an oxidative degradation of the protein during inflammation [26] rather than disturbances of copper levels. As CuZnSOD is located intracellular in ciliated epithelium [28], any disturbance in copper levels and CuZnSOD can result in further progression of oxidative stress.

Another interesting finding has been described by Liu et al. [29]. They found a decreased level of copper after chronic tumor necrosis factor-alpha (TNF-alpha) dependent lung inflammation as well as in TNF-alpha overexpressing mice. This suggests that copper ions play a role in inflammation-induced lung damage and that there is a link between levels of TNF-alpha and copper. This in turn points that copper can be seen as a factor in disease progression and that dietary copper deficiencies affect immunity. The latest findings about the importance of metals such as copper, iron, and zinc in immunity have been reviewed by Healy et al. [30].

### 2.2. Selenium

There are many excellent papers reviewing and explaining the role of selenium in immunity and asthma [31,32,33,34,35,36,37], and it is impossible to describe in few lines the impact of selenium and selenoproteines in various aspects of immunity, inflammation, and inflammatory disorders. In short, selenium (Se) is a micronutrient strongly involved in the antioxidant defense mechanisms of the body as a cofactor of glutathione peroxidase (GPx), one of the most important antioxidants, thioredoxin reductase, and iodothyronine deiodinases, a class of enzymes controlling the proper levels of thyroid hormones [31]. The link between asthma and selenium is complex and relies not only on the interplay between selenium content, GPx activity, and oxidative stress (according to Carneiro et al. [38], low selenium levels lead to a strong decrease of antioxidant protection) but also on the influence of selenium on the Th1/Th2 balance [35]. It is believed that selenium promotes T cell differentiation towards Th1 via an increase of Th1 cytokine expression and/or by inhibition of Th2 cytokine secretion [39,40]. In this context, Guo et al. [15] note that increased inflammation leads to a decrease in selenium absorption and, thus, a decreased selenium status. However, reduced Se intake itself can also lead to a decreased selenium status in the serum of asthmatics [41]. However, the reason asthmatics have lower Se intake is not known.

There is a strong agreement that asthmatic subjects have lower Se levels [7,9,14,38,42,43,44,45,46,47], lower GPx activity [7,44,48] and that Se deficiencies are associated with increased risk of asthma by up to five times [48], respiratory symptoms, and worse pulmonary function [38,43,48,49,50,51,52,53]. A meta-analysis performed by Chen et al. [54] showed that in the general population, lower Se levels are associated with higher asthma risk. Baiz et al. [55] and Devereux [49] observed that higher maternal Se serum concentration decreases the risk of wheeze but not of fully symptomatic asthma in children up to the age of 5. Parallel, Shaheen et al. [56] mention that suboptimal Se in maternal serum leads to suboptimal GPx function and impaired antioxidant mechanism against oxidative stimuli in fetal airway epithelium, which, in turn, leads to epithelial damage and contributes to asthma development. This was also proposed by Broome et al. [39] and Devereux et al. [49] who point that a disturbed antioxidant status during early life leads to an increased Th2 polarization, which increases pro-inflammatory status as well as the risk of atopic sensitization.

In contrast, some researchers [57,58,59,60] could not find any association between selenium levels and asthma, even if a decrease of GPx activity was observed [61]. Additionally, both Nwaru et al. [62] and Martindale et al. [63] could not find any relationship between maternal selenium intake and prevalence of asthma in the offspring. However, both papers did not mention any additional data on mineral supplementation and on feeding habits during the first years of life of the child. It is possible that pre-natal deficiencies have been corrected during early life without any further consequence.

In most cases, selenium supplementation reverses the decreased antioxidant parameters, as has been described by Guo et al. [7], Baker et al. [64], Fabian et al. [46], and Hasselmark et al. [65] who observed a general clinical improvement of asthma. Gazdik et al. [66] found in their study that a restoration of the proper selenium status leads to a decrease in use of oral and inhaled corticosteroids in asthmatic patients. Moreover, a better lung function of asthmatics after Se supplementation has been observed [50] together with a lower prevalence of asthma in the general population [51], wherein Se supplementation decreased the risk of asthma by 20% in children and even by 50% in a population of passive-smoking children. On the contrary, Shaheen et al. [67] could not find any clinical benefits of Se supplementation in adult asthmatics.

In animal models, Safaralizadek et al. [68] have observed that Se supplementation leads to a decreased release of prostaglandin D2 and histamine from activated murine mast cells. In another experiment performed by Jeoug et al. [40], pretreatment with selenite (a selenium salt) prior to ovalbumin (OVA)-sensitization in a murine allergic asthma model, abolished the increase of adhesion molecules and cellular influx and increased the activity of selenium-dependent GPx in lungs. Jiang et al. [69] supplemented selenium during OVA-sensitization and found a strong decrease of interleukin (IL)-4, 5, 13, 25, and 33 with no effect on total IgE or histamine levels. In another cockroach murine model of allergic asthma, Bansal et al. [70] observed, after Se supplementation during sensitization, a decrease of cellular influx into the airways, decrease of serum IgE, proinflammatory cytokines such as IL-4 and 5, and increase of the anti-inflammatory IL-10 in bronchoalveolar lavage fluid (BALF) together with an increase in GPx activity in lung tissue. However, no changes in airway hyperreactivity have been noted.

However, selenium supplementation can, in some cases, worsen asthma outcomes as it has been presented by Hoffmann et al. [71,72]. The authors point out that selenium can have a double role and that a low-Se diet before the induction of asthma in a murine OVA model can lead to lower susceptibility to the disease whereas high-Se diet can worsen the disease. They found that the higher the Se supplementation was, the higher was the activity of GPx but also the inflammatory cell count in BALF, goblet cell hyperplasia, the level of anti-OVA IgE and airway hyperreactivity. These observations might be related to the selenium content in the diet which could in the latter group significantly exceed the organism’s needs.

Taken together, selenium plays an essential role in immunity and defense against oxidative stress. Nonetheless, as with every metal taking part in the anti-oxidative protection including copper, iron, and zinc, its excess can lead to a shift towards a more pro-oxidant state.

### 2.3. Zinc

Zinc (Zn) is, together with selenium, probably the most widely described micronutrient in asthma and other inflammatory diseases (for details see the excellent reviews by Devirgiliis et al. [10], Mohamed et al. [73], Suzuki et al. [74] and Zalewski et al. [75]), and in the current paragraph only the most important findings are presented. Zinc is the second most abundant trace metal in the mammalian body after iron. It is, together with selenium, one of the most important factors keeping the correct oxidant-anti-oxidant balance of the organism, and as a consequence, the proper immune reactions. A detailed mechanism of antioxidant properties of zinc has been described by Prasad [76]. In short, zinc diminishes oxidative stress by inhibiting reactive oxygen species (ROS) production via inhibition of pro-oxidant enzymes, such as NADPH oxidase and iNOS, and by activation of anti-oxidant enzymes including glutathione-related enzymes, catalase (CAT) and SOD. Immunomodulatory features of zinc and the mechanisms underlying its action have been presented in details by Zalewski et al. [75] and Suzuki et al. [74]. It seems that the most important functions of Zn ions are restoration of the impaired balance between Th1 and Th2 cells, and excessive production of pro-inflammatory cytokines both being the main features of asthma.

Zinc deficiencies are quite common in humans. Their clinical symptoms include rough skin, poor appetite, recurrent infections of the respiratory tract, weight loss, poor darkness adaptation, diarrhea, neurological and emotional disorders, etc. [77,78]. Zinc deficiencies are found in inflammatory diseases [10,75] including asthma, where low zinc levels are risk factors of development, severity, and exacerbations of asthma [10].

First of all, zinc is not stored by the body, so a regular dietary or supplement intake is needed. There are many reports showing that proper levels of zinc and other anti-oxidants and higher maternal intake of this element during pregnancy prognoses better pulmonary functions of the offspring and lower risk of developing asthma in childhood [49,79]. Low zinc intake increases (up to five times) the risk of developing atopy, bronchial reactivity, and allergy-like symptoms [10,80]. Zinc deficiencies have been reported in many disorders, including asthma. There are many reports on the levels of zinc in asthmatic subjects. Most of them show a decrease of zinc concentration in the serum or induced sputum of asthmatic adults [14,22,73,77,79,81], and children [82,83,84,85]. Siripornpanich [86] in Thailand and Khanbabaee [84] in Iran found zinc deficiency in the serum of asthmatic children together with increased oxidative stress and airway inflammation. They also found a correlation between zinc levels and breathing parameters such as forced expiratory volume in 1 second (FEV1) and forced expiratory volume in 1 second to forced vital capacity ratio (FEV1/FVC) and concluded that zinc deficiencies are related to severe asthma and to decreased pulmonary function. In some reports such deficiencies could not be found in erythrocytes of asthmatic and healthy children [87,88] or in plasma of asthmatic adults [89]. Soutar et al. [80] describes that lower zinc levels lead to an increased risk of asthma attacks.

Most of the symptoms of zinc deficiency can be easily reversed by zinc supplementation as it has been described by Prasad et al. [78] who found that Zn supplementation diminished the number and symptoms of respiratory tract infections.

The main mechanism underlying the immunomodulatory and anti-inflammatory action of zinc is the maintenance of the correct balance of Th1/Th2 cells. Zinc deficiency, as well as depletion of glutathione (GSH), lead to a shift towards Th2 [90] and to an induction of pro-inflammatory mediators including cytokines IL-4, 6, leukotriene B4 (LTB4) and prostaglandin E2 release. Zinc restores the proper Th1/Th2 balance [90,91], enhances the overall antioxidant natural deference [92], and reduces the propagation of inflammation by inhibition of LTB4 production and expression of its receptors [90].

Another zinc-dependent class of enzymes whose functions are altered in asthma are matrix metalloproteinases which are, among others, involved in the collagen turnover and physiologic remodeling processes. It is unclear whether zinc deficiency leads to the abnormal airway remodeling by dysregulation of matrix metalloproteinases, however, Xu et al. [93] showed that decreased zinc levels enhance fibrosis in experimental kidney fibrosis.

At the level of the airway epithelium (for details about the importance of zinc for the proper functioning of airway epithelium, see [75]), Zn deficiency makes it more susceptible to damage and to cell apoptosis both in vitro [94] and in vivo [95,96] and, as a consequence, worsens airway inflammation [97]. Also, increased inflammation and airway hypersensitivity has been observed [96].

At the cellular level, Tsai et al. [98] showed that peripheral blood mononuclear cells (PBMCs) grown in Zn supplemented media released less Th2-cytokines including IL-4 and more Th1 ones such as interferon gamma (IFN-gamma), which confirms again that zinc supplementation leads to a correction of the impaired Th1/Th2 shift. Interestingly, mild Zn deficiency leads to an increase of IL-1beta level in monocytes, one of the cytokines promoting airway hyperreactivity [76].

An interesting finding has been described by Morgan et al. [99] on a mice model of allergic exposure. Zn administration prior to allergy induction leads to decreased neutrophil infiltration and lower TNF-alpha release into the airways together with decreased NF-kB activity in the lungs. Moreover, Zn supplementation after allergen exposure decreased airway hyperreactivity and IgE levels. Additionally, Lang et al. [100] observed a decrease in eosinophil and lymphocyte influx into the airways of allergic asthmatic mice, which has been confirmed by Lu et al. [101] who described an increased eosinophil, neutrophil and monocyte count as well as increased eotaxin and monocyte chemoattractant protein-1 (MCP-1) levels in BALF of OVA-sensitized zinc-deficient rats. Once again, Zn supplementation suppressed inflammatory cell infiltration and pro-inflammatory protein secretion, leading to the conclusion that restoration of zinc levels may lessen the severity of inflammation. Seo et al. [78] found that zinc supplementation inhibits mast cell degranulation leading to a decrease of histamine production.

Zinc supplementation can not only prevent asthma but also decrease the severity of asthma exacerbations and lead to a faster resolution of asthma attacks [102]. A randomized, placebo-controlled study on Iranian asthmatic children treated with inhaled corticosteroids showed that eight-week zinc supplementation decreased wheezing, cough, and dyspnea, and ameliorated lung parameters such as FVC, FEV1, and FEV1/FVC ratio.

To conclude, proper zinc levels are essential for the maintenance of the balance of pro- and antioxidative as well as pro-and anti-inflammatory responses which are disrupted in asthma. It seems that zinc supplementation can partially restore the proper functioning of the asthmatic airway and in a part diminish its symptoms leading to a better asthma control.

## 3. Micronutrients Affecting the Main Risk Factors of Asthma

### 3.1. Chromium

There are doubts concerning whether chromium should be seen as an essential trace element. Little is known about the biological functions of the element, and it is still under discussion if chromium deficiencies are present in humans and, if yes, in which disorders. There is evidence for the effectiveness of chromium supplementation in improving some symptoms of the metabolic syndrome and diabetes type 2 in patients, but there are no effects in case of healthy individuals. Chromium ions are present in the human body, and there are special carrier proteins for this element—chromodulins which interact with the insulin receptor and improve glucose absorption [103].

Chromium is mostly known for two contradictory features, depending on its chemical status. Chromium at the oxidative state (+6) is irritant, toxic, and mutagenic when inhaled or ingested [104,105]. It is one of the most important causes of occupational asthma in workers [106], and together with nickel, one of the major causes of metal-dependent contact dermatitis [107]. This is related to the fact that hexavalent chromium ions are strong oxidizers, and thus increase oxidative stress and induce inflammation [108]. They can also penetrate, contrary to Cr3+ ions, through cell membranes and induce intracellular oxidative stress by being reduced directly within the cell [109]. Cr (+6) also aggravates the symptoms of asthma [110] and other lung diseases [111].

Chromium at the oxidative state +3 takes part in regulation of energy metabolism. It is believed to enhance lipid and hydrocarbonate turnover [103], together with stabilization of the number of insulin receptors and in general increasing insulin sensitivity [112,113]. However, it is supposed that a beneficial action of chromium supplementation would occur only in case of its well pronounced deficiencies, such as in obesity and insulin-related disorders including the metabolic syndrome [114].

Chromium (3+) acts as an antioxidant by increasing the levels of reduced glutathione, SOD and catalase activity [115,116].

Up to now, studies on the influence of chromium on manifestations of respiratory diseases are scarce. The only study on the influence of chromium supplementation on respiratory functions was performed by Kolahian et al. [117] on diabetic rats. It has been found that chromium supplementation decreased lung and bronchi inflammation and macrophage influx to the alveoli, increased antioxidative capacity, and decreased oxidative stress in both blood and lungs. All of these symptoms are present not only in diabetes but also in asthma. In consequence, chromium supplementation could be beneficial in alleviating asthma symptoms, not only directly by correcting the impaired glucose metabolism in obesity-related forms of asthma, but also by its anti-inflammatory and antioxidant properties. However, extensive research on asthma symptoms and chromium supplementation is still needed.

### 3.2. Iodine

Contrary to fluoride (see below), iodine and its disturbances play a role in asthma pathology. Iodine is a component of the iodinated thyroid hormones: triiodothyronine (T3) and thyroxine (T4), both regulating various metabolic pathways including protein synthesis, glucose and fat turnover, and thermogenesis. In general, iodine deficiency leads to hypothyroidism with low ability to tolerate cold, tiredness, and weight gain as symptoms, whereas excess of iodine can induce hyperthyroidism with nonspecific such as weight loss, emotional labiality, nervousness, irritability, tachycardia, and muscular weakness. In susceptible patients with previous thyroid disorders, iodine overload can lead to one of the two opposed morbid states, namely iodine-induced hypothyroidism or iodine-induced hyperthyroidism (as it is discussed by Leung and Braveman [118]).

It is generally accepted that there is a metabolic link between disturbances of thyroid hormones and asthma. Fekri et al. [119] described in their research that asthmatic women have higher levels of anti-thyroid peroxidase than non-asthmatic ones and free T4 levels may be a determinant of refractory asthma [120,121]. Already during the prenatal period, maternal hypothyroidism increases the risk of asthma in the offspring [122,123] and impairs lung development [122,124]. In both children and adults, increased thyroid activity (hyperthyroidism) worsens asthma. There is an increased incidence of goiter in asthmatic patients, and hyperthyroid patients tend to have lower lung parameters including vital capacity [125]. The observed deterioration of asthma symptoms may be related to an increased production of reactive oxygen species and elevated oxidative stress in hyperthyroid patients, which can be partially reversed by thyroid-stabilizing treatment [125]. It seems that the pro-inflammatory effects of thyroid hormones are mediated by a direct or indirect (via T cells) activation of B lymphocytes [126]. Dekkers et al. [127] described that thyroxine (T4) increases the proliferation of airway smooth muscles and as a consequence enhances airway smooth muscles remodeling leading to an increased airway hyperreactivity and aggravation of asthma symptoms. Moreover, high serum levels of T4 inversely correlate with response to beta2 adrenoreceptor agonists, which in turn hamper asthma treatment [128]. It seems that pro-inflammatory cytokines such as TNF-alpha inhibit the regulatory activity of the thyroid-stimulating hormone (TSH) and suppress the conversion of T4 to T3 [121], increasing inflammation.

On the other hand, the presence of hypothyroidism improves asthma outcomes but a correction of thyroid status leads to a restoration of asthma symptoms including airway hyperreactivity and inflammatory cell count [125,129]. In Chinese population of old male asthmatics Bingyan et al. [121] observed a decreased level of both total and free T4 and T3 hormones during exacerbations. They also found that breathing parameters such as FEV1 and FEV1/FVC were correlated with levels of free thyroid hormones. In 1999 Manzolli et al. [126] induced allergic asthma in an OVA model in rats 50 days after surgical thyroidectomy. They found a strong decrease in cellular influx into the airways together with a decreased level of IgE antibodies against OVA; thyroid-depleted rats showed a lower weight gain and lower serum levels of thyroid hormones. All of these symptoms were reversed after a prolonged, 16-day treatment with T4. Interestingly, when OVA sensitization was performed before surgery, the animals showed regular asthma symptoms including elevated cellular influx. These results show that thyroid hormones are capable of controlling asthma via production of IgE antibodies.

The use of thyroid hormones and iodine in treatment of asthma is controversial [121] even if lecithin-bound iodine (LBI) has positive effects in allergic asthma as it was described by Kawano et al. [130,131]. LBI corrected abnormal mite-allergen induced immune responses in peripheral blood mononuclear cells by inhibition of production of IgE antibodies and IL-4 in both asthmatics and controls. This beneficial action on asthma symptoms is supposed to be mediated by a restoration of the proper balance of Th1/Th2 cytokines. At the same time, LBI decreases iodine-dependent goiter [130,131] regulating the activity of the thyroid gland.

### 3.3. Iron

The first connotation between asthma and iron is oxidative stress. Iron is the main catalyst of the Fenton reaction, one of the sources of free radicals (reactive oxygen and nitrogen species, ROS and RNS) formation. Ghio [132] even titled his review on the links between iron and risk factors for asthma exacerbations *“Asthma as a Disruption in Iron Homeostasis”.* However, only free iron takes part under standard conditions in the formation of ROS, and thanks to the omnipresence of iron-binding proteins, free iron levels remain strictly controlled. The iron cycle, its impact on oxidative stress, and iron-binding, releasing and transporting proteins have been presented in detail by Turi et al. [133]. In brief, there are four major iron binding proteins which are of clinical value: lactoferrin with immunomodulatory properties, ferritin, the major iron storage protein, ferroportin, a transmembrane iron transporter, and transferrin, the main plasma iron carrier [134]. All of them seem to protect lung tissue from oxidative stress, whereas free iron is its inducer. As it has been described by Ali et al. [135], dysregulations of iron status and iron homeostasis occur in many diseases including diseases of the respiratory system. In the respiratory tract, iron-related proteins are secreted by epithelial cells, macrophages (described by Ganz [136] and Gammella et al. [137] as the main iron-regulating system), and neutrophils to control free iron levels under physiological conditions.

As it is in case of other micronutrients, there are contradictory findings about the relationship between iron levels and asthma. In most studies serum iron levels were lower when compared to healthy subjects [138,139,140]. The link between low serum iron levels and chronic inflammation is missing even if short iron supplementation decreases airway inflammation in acute asthma animal models [141]. Other studies [9,142,143] showed increased plasma levels of iron together with increased oxidative stress markers. At the same time, Vlasic et al. [140] found in exhaled breath condensate of asthmatic children decreased levels of iron together with increased activity of SOD indicating oxidative stress, and fractional exhaled nitric oxide (FeNO) confirming the presence of airway inflammation. Similarly, Ali et al. [144] found decreased cell-free iron levels in BALF of asthmatics together with an increase in the number of iron-loaded cells. These findings correlated with lower FEV1/FEC index.

An interesting finding was described by Guo et al. [15] who found increased hemoglobin levels in asthmatics, due probably to increased oxygen demand.

Rhew et al. [145,146] showed that the prevalence of anemia and, as a consequence, low iron status was higher in atopic (suffering from atopic dermatitis, allergy and asthma) patients in Korean children and the general population. The same was showed by Drury [147] in the US. This phenomenon might be due to that atopic diseases involve inflammation and these, in turn, can cause iron deficiency. Moreover, Brigham et al. [138] showed that higher iron levels decreased the risk of asthma. This was confirmed in an OVA murine model of asthma by Maazi et al. [141] who found that both dietary and systemic iron supplementation decreased eosinophilia and airways hyperresponsiveness. Coming back to humans, Brigham et al. [138] described that high serum ferritin levels, as a reliable measure of iron store of the organism, decrease the risk of asthma. At the same time, high iron needs, described as high transferrin levels, are linked to lower lung parameters including FEV1. That leads to the conclusion that low iron levels together with high iron need impairs lung function. These findings could as a consequence, explain the increased prevalence of asthma during menstruation [132].

An interesting issue is the impact of iron chelation on asthma symptoms. Bibi et al. [148] showed that an iron chelator based on zinc-/gallium-complex with desferrioxamine decreased most of the asthma features of allergic asthma in mice, including cellular influx, number of inflammatory cells and mucus secretion. Furthermore, desferrioxamine, an iron chelator, alone has anti-inflammatory properties as it was presented by He et al. [149] who found that chelation of iron by this compound strongly decreased eosinophilia in a lipopolysaccharide (LPS)-induced exacerbation of OVA-induced allergic asthma in mice. This in turn leads to the conclusion that free iron takes part in asthma exacerbations.

Lactoferritin (LFT) has similar properties related to its ability to bind iron. LFT, found in mucosal secretion or neutrophilic granules, prevents systemic inflammation, decreases oxidative stress, lipid peroxidation, and production of pro-inflammatory cytokines [150,151]. These findings were confirmed by Bournazou et al. [152] who found that LTF decreased airway inflammation and inflammatory cell influx in a murine model of allergic asthma, all of them being due to iron chelation by LTF.

There is a link between maternal iron status and lung condition of the child. There are many studies [56,153,154,155,156], showing that low maternal iron status leads to an increased risk of wheezing, atopy and impaired lung functions including FVC and FEV1 of the child. It seems that routine iron supplementation during pregnancy reduces the risk of asthma in the offspring [56,157]. Nwaru et al. [153] and Quezada-Pinedo et al. [156] link these findings to the fact that lower maternal iron status impairs the correct lung development of the fetus, decreases the proper oxygen supply, and results in worsened lung growth and lower lung function of the child.

To sum up, as it was concluded by Ali et al. [144], iron, especially when elevated, plays a key role in the pathogenesis and severity of asthma, and proper levels not only of iron, but also of iron-stabilizing proteins are needed for asthma control.

### 3.4. Manganese

Manganese (Mn) is another micronutrient taking part in the protection against oxidative stress in asthma. First of all, it is the main component of one of the forms of superoxide dismutase (SOD) (MnSOD) located in the bronchial epithelium, alveolar type II epithelial cells and alveolar macrophages, mostly in mitochondria [28]. MnSOD is activated by pro-inflammatory cytokines such as TNF-alpha [158] and inactivated by anti-inflammatory agents such as dexamethasone [28]. Most of the actions of Mn on the respiratory system are connected to the antioxidant defense system.

There are contradictory reports about the level of Mn in asthma. Huang et al. [159] found decreased levels of this element in the urine of asthmatics, as did Oluwole et al. [160] in the serum of Nigerian children. Gray et al. [11] found increased Mn levels in sputum whereas Mutti et al. [161] could not find any differences in Mn content in exhaled breath condensate between asthmatic and healthy subjects, as did Urushidate et al. [60] in serum and Moresco et al. [16] in nails. Nevertheless, Patel et al. [162] observed lower intake of Mn by asthmatics and it seems that low Mn intake is associated with increased bronchial responsiveness [79,162] and up to five-time increased risk of asthma [41,80]. At the same time, Patel et al. [162] pointed out that Mn levels and dietary Mn intake influence MnSOD activity. There are no conclusive data about the influence of maternal intake of Mn on asthma or wheeze in children. Both Nwaru et al. [62] and Martindale et al. [63] could not find any association between these two parameters.

Kocygit et al. [8,9] proposed an interesting theory about the relationship between Mn and asthma. Decreased Mn levels would lead to decreased arginase levels and, as a consequence, to increased NO levels, as it is observed in asthma [163]. As arginase and nictric oxide synthase (NOS) compete for L-arginine, and NOS activity is increased in asthma [164], a proper level of arginase would limit the arginine substrate for NO synthesis and, thus, limit the NO levels (and furthermore asthma symptoms). As a consequence, proper Mn levels could alleviate, at least partially, some asthma features. This is in line with the findings of Terziev et al. [165], Cao et al. [166], and Chang and Crappo [167,168] who state that Mn-related antioxidants could mitigate the effects of oxidative stress in asthma. They found that a Mn-porphyrin complex (an anti-oxidant mimetic) increased SOD and CAT activity in a murine OVA-model of asthma, decreased airway inflammation, airway hyperreactivity, inflammatory cell count in BALF as well as the expression of adhesion molecules. These findings indicate that Mn supplementation increases the antioxidant features of the body and alleviates most of the asthma symptoms.

## 4. Micronutrients of Minor or Unknown Influence on the Course of Asthma

### 4.1. Boron

The biological role of boron is not well understood. It seems that boron, together with calcium, magnesium, and vitamin D, regulates bone tissue metabolism and its deficiencies lead to calcium lost and demineralization of bones [169]. Moreover, boron supplementation alleviates the outcomes of magnesium, calcium, and vitamin D deficiencies [170]. It also stabilizes insulin release and corrects impaired lipid metabolism [171].

There is a connection between boron, the immune system, and inflammation. Boron supplementation reduced the severity of arthritis symptoms such as joint rigidity, diminished mobility, or pain [172]. In vitro, boron supplementation decreased the production and release of IL-1beta and IL-6 from stimulated macrophages [173] and THF-alpha from LPS-stimulated monocytes [174].

Little is known about the deficiencies of boron in asthma and other inflammatory disorders of the respiratory system. There are several factors pointing such a possibility, including a correct dietary level of boron alleviates consequences and symptoms of magnesium and vitamin D deficiencies, both of these having been reported in the course of asthma [175]. Moreover, boron shows anti-inflammatory properties and regulates steroid hormone-depended disturbances in immunity.

Boron acts as an anti-inflammatory via several pathways. First, its supplementation increases the levels of antibodies after vaccination with typhoid vaccine in rats [176], probably by a stabilization of function of T-cells (which is also disordered in asthma). Another issue connected to the role of boron in asthma pathophysiology is the fact that boron supplementation increases the levels of steroid hormones by inducing 17-beta hydroxylation leading to an increase of estrogen in rats [177] and in postmenopausal women [178]. It has been discovered that menopause, as well as other disruptions of production of steroid hormones, are risk factors for a late onset of asthma, as a decrease of estrogen levels increases the production of pro-inflammatory cytokines [179]. Also, the estrogen-dependent path of T-cell regulation seems to be influenced by boron and its correcting action towards steroid hormones.

Boron supplementation regulates the natural redox-balance which is disturbed in asthma. This occurs involving several systems, including glutathione (as boron inhibits the gamma-glutamyl-transpeptidase (GGT), leading to an increase of the GSH content [180]) and the activity of SOD (by increasing its concentration in erythrocytes [181], and by inhibiting the lipoxygenase system [182]).

However, even if there are presumptions that boron deficiency might take part in the pathophysiology of asthma and its supplementation might alleviate its symptoms, up to now there have been no scientific reports or research articles on this subject.

### 4.2. Cobalt

Up to now, there are no data on cobalt deficiencies in respiratory diseases in humans or in rodents. Deficiencies of this metal are rather related to deficiencies in vitamin B12 (cobalamin) levels, in which the cobalt ion is the active side. Vitamin B12 plays a role in blood cell formation, DNA synthesis, synthesis of myelin, and normal functioning of the nervous system [183]. Despite the fact that cobalamin acts as an immunomodulator [184], no associations between its levels and the prevalence of asthma or allergy could be found [185,186,187].

### 4.3. Fluorine

There are no reports on direct or indirect links between fluorine deficiency or overload and asthma. Fluorinated compounds have been used in treatment of asthma [188]. In these cases, however, fluorine is covalently bound to the molecule. As ions, fluorides decrease airway hyperreactivity and show bronchodilating activity in asthmatics during the methacholine test, as it has been described for sodium fluoride by Zhao et al. [189]. It seems that this phenomenon occurs by the inhibition of enolase, one of the enzymes of the glycolysis pathway by fluoride anions.

Fluorine deficiencies are often associated with an increased risk of caries and other dental diseases. Gani et al. [190] summarized that a higher incidence of caries in asthmatics than in healthy subjects has been observed. However, these problems in asthmatics are rather associated with the use of medications and lower saliva flow, both leading to disturbances in oral microbiota, and no interplay between dental fluoride status and asthma could be found.

### 4.4. Molybdenum

One of the less known micronutrients is molybdenum (Mo). Mo is the main constituent of the so-called molybdenum cofactor (Moco) which, in turn, is the main active side of such enzymes such as xanthine oxoreductase (XOR) or aldehyde oxidase, as well as of many plant enzymes. Although molybdenum deficiencies are rare and lead to renal failure, a proper level of Mo (and, as a consequence, of Moco and XOR) has to be maintained. XOR catalyses not only the last step of purine degradation but generates the superoxide anion radical and, thus, under specific conditions takes part in ischemia-reperfusion injury and transduction of the inflammatory signal [191,192]. There is little known about XOR in the respiratory system. Battelli et al. [193] cited that under inflammatory conditions including stimulation of lung epithelial cells by pro-inflammatory cytokines, hypoxia in acute lung injury, or pneumonia, XOR expression increases whereas hyperoxia decreases its activity. There are only two reports dealing with the importance of XOR and Mo in asthma. In the first, Huang et al. [159] described an increased level of Mo in the urine of asthmatic patients without changes in lung function. In the second, Setiawan et al. [194] found in a murine house dust mite model of allergic asthma increased production of the superoxide anion radical from XOR in lungs without changes of its expression. However, an increased expression of XOR together with an increased H2O2 content was observed in BALF. Interestingly, no XOR could be detected in bronchial epithelium of asthmatic mice. This leads the authors to the conclusion that in asthma XOR is moved from bronchial epithelial cells to the airway lumen, and thus, takes part in the defense mechanisms of asthma. Therefore, it seems that Mo and its deficiencies have little or no direct influence on the course of asthma.

## 5. Summary

Micronutrients play a pivotal role in immunity and protection against oxidative stress. Some of them do not have a direct role in the pathogenesis of asthma, as it is in the case of boron, fluorine, or molybdenum; some play an indirect one such as iodine or chromium. Others, including copper, iron, manganese, selenium, and zinc are indispensable in the correct defense mechanisms, and disturbances of their levels, both too low and too high, or of their incorporation into enzymes lead to perturbations in the proper functioning of the body (as it has been summarized in Table 1). This is of highest importance in the case of asthma, which pathophysiology relates to an imbalance of the redox state (as oxidative stress) [195], or improper functioning of the antioxidant systems [196], and local and partially systemic inflammation. More important is the restoration of proper levels of micronutrients, with special consideration of the particular needs of a diseased organism, to partially alleviate the symptoms of one of the most prevalent diseases of the modern world, namely asthma.

Even if, as it has been discussed above, there are many reports on the importance of micronutrients in asthma, there is only limited evidence that the findings could be directly translated into clinical practice. Formulations with vitamins and minerals are rarely drugs undergoing the entire cycle of registration and are in most cases only dietary supplements which are subject to less strict legal requirements. As a consequence, access to them is much easier; they can be purchased everywhere, and there is no control on their intake and composition. Moreover, many physicians are not aware of the importance of micronutrients and do not prescribe supplementation, leaving the choice to the patients without giving exact dosage or composition. Thus, there is a long way from animal studies and clinical observations to everyday practice.

One might argue if micronutrient supplementation could prevent asthma and asthma-related symptoms. This seems to be rather less probable as there are many risk factors underlying this complex disease. Another problem is that it is still not known if the observed deficiencies of micronutrients are primal or secondary to asthma and asthma treatment as it has been discussed by Zalewski et al. [73]. Nonetheless, correct levels of micro-and macronutrients enable proper defense mechanisms and general functioning of the organism leading to a lower vulnerability to diseases.

This review has its obvious limitations. First, this review is a summary of the present knowledge on micronutrients in asthma which are (as in case of zinc or selenium) or are not yet (as with molybdenum or fluorine) widely investigated. Consequently, not much information could be included about some minerals. Second, this paper aims to be a narrative review and no further analyses of data have been performed. Still, it shows which minerals and their influence on the course of asthma remain unexplored and where further research is still needed to understand the less obvious processes underlying asthma and asthma-related symptoms.

## Figures and Tables

**Table 1:** Summary of the role of micronutrients and their disturbances in asthma.

Micronutrient	Biological Action	Levels in Asthma	Possible Mode of Action in Asthma
Copper	-Co-factor of enzymes including cytochrome c oxidase, Cu-Zn-superoxide dismutase [26]; -Takes part in iron metabolism [13]	Increased in asthma [11,12,13,14]	Overload: -Disturbances in redox balance and control of oxidative stress [15]; -Disturbances of collagen cross-linking leading to airway remodeling [24]
Selenium	-Co-factor in glutathione peroxidase and other antioxidant enzymes [31,32,33,34,35,36,37]; -Takes part in iodine metabolism [31]	Decreased in asthma [7,9,14,38,42,43,44,46,47,54]	Deficiency: -Disturbances of redox balance and control of oxidative stress [7,44,45]; -Decreased immunological response [31,32,33,34,35,36,37]
Zinc	-Co-factor of Cu-Zn-superoxide dismutase, metalloproteinases [76,93]; -Maintenance of Th1/Th2 balance [74,75]; -Control of propagation of inflammation [90]	Decreased in asthma [14,22,73,77,79,81,82,83,84,85,86]	Deficiency: -Disturbances of redox balance and control of oxidative stress [84,86]; -Increased inflammatory responses [96]; -Enhanced fibrosis [93]
Chromium	-Takes part in energy metabolism [103]; -Controls indirectly glucose and insulin levels [103]; -Acts as an indirect anti-oxidant by increasing the activity of antioxidant enzymes [115,116]	Unknown, lack of studies	Deficiencies: -Increased inflammation and oxidative stress [117]; -Poor obesity-dependent asthma control and diseases progression [114] Note: Used in obesity control, one of the risk factors for asthma
Iodine	-Control of thyroid gland and hormones [118]	Both deficiency and overload observed in asthma and thyroid-dependent metabolic disorders influencing indirectly the course of asthma [119,120,121,122,123,124,125,129]	Deficiency and overload: -Impaired inflammatory responses [126]; -Worsening of asthma outcomes [127]
Iron	-Takes part in oxygen transport [15]; -Control of oxidative stress [133]	Both deficiency (anemia) and overload [138,139,140,142,143,145,146]	Deficiency: -Worsening of asthma outcomes [140] Overload: -Increased oxidative stress and inflammation [144] Note: The most important is the maintenance of proper levels of iron-binding and -stabilizing proteins
Manganese	-Co-factor of Mn-superoxide dismutase [28]	Inconclusive data [159,160,161]	Deficiency: -Increased inflammation and oxidative stress [167,168]; -Worsening of asthma outcomes [79,162]
Boron	-Takes part in bone tissue metabolism [169]; -Interacts with the immune system [172]	Not known, lack of data	Possible consequences of deficiency: -Increased inflammation [173,176]; -Increased oxidative stress [180,181,182]
Cobalt	-Co-factor of vitamin B12 [183]	No association found [185,186,187]	Possible immunomodulator [184]
Fluorine	-Takes part in teeth formation and maintenance [189]	Unknown, lack of data	Unknown
Molybdenum	-Co-factor of xanthine oxidase [191,192]; -Transduction of inflammatory signal [191,192]	Scare data, rather increased [159]	Overload: -Increased oxidative stress or no effect [194]
